# Taxonomic and phylogenetic characterizations reveal four new species of *Simplicillium* (Cordycipitaceae, Hypocreales) from Guizhou, China

**DOI:** 10.1038/s41598-021-94893-z

**Published:** 2021-07-27

**Authors:** Wan-Hao Chen, Yan-Feng Han, Jian-Dong Liang, Zong-Qi Liang

**Affiliations:** 1 Basic Medical School, Guizhou University of Traditional Chinese Medicine, Guiyang, 550025 Guizhou People’s Republic of China; 2 Department of Ecology, Institute of Fungus Resources, College of Life Sciences, Guizhou University, Guiyang, 550025 Guizhou People’s Republic of China

**Keywords:** Fungal systems biology, Fungal evolution

## Abstract

*Simplicillium* species are commonly found from soil, seawater, rock surface, decayed wood, air and as symbiotic, endophytic, entomopathogenic and mycoparasitic fungi. Minority insect-associated species was reported. *Simplicillium coccinellidae*, *S. hymenopterorum*, *S. neolepidopterorum* and *S. scarabaeoidea* were introduced as the newly insect-associated species. The phylogenetic analyses of two combined datasets (*LSU* + *RPB1* + *TEF* and *SSU* + *ITS* + *LSU*) revealed that *S. coccinellidae* and *S. hymenopterorum* were both nested in an independent clade. *S. neolepidopterorum* and *S. scarabaeoidea* have a close relationship with *S. formicidae* and *S. lepidopterorum*, respectively. *S. neolepidopterorum* can be easily distinguished from *S. formicidae* by ellipsoidal to cylindrical, solitary conidia which occasionally gather in short imbricate chains. *S. scarabaeoidea* could be easily distinguished from *S. lepodopterorum* by having longer phialides and larger conidia. Based on the morphological and phylogenetic conclusion, we determine the four newly generated isolates as new species of *Simplicillium* and a new combination is proposed in the genus *Leptobacillium*.

## Introduction

The genus *Simplicillium* was established for the typical species *S. lanosoniveum* (J.F.H. Beyma) Zare & W. Gams and three other species *S. obclavatum* (W. Gams) Zare & W. Gams, *S. lamellicola* (F.E.V. Sm.) Zare & W. Gams and *S. wallacei* H.C. Evans^1^. The typical characteristic of *Simplicillium* is its solitary phialides, which could be easily distinguished from its closely genus *Lecanicillium* W. Gams & Zare. *S. wallacei* was transferred to the genus *Lecanicillium* based on the phylogenetic analysis by Zare & Gams^2^. Fourteen species were reported later. Okane et al.^3^ transferred *S. chinense* F. Liu & L. Cai and *S. coffeanum* A.A.M. Gomes & O.L. Pereira to the genus *Leptobacillium* and this transfer was confirmed by Wang et al.^4^.

*Simplicillium* species have diverse ecology, but most species are known from few strains impeding to define their habitat and ecology accurately. Species were found from soil (e.g., *S. cylindrosporum*, *S. minatense*, *S. subtropicum*, and *S. sympodiophorum*^5^), as plant endophyte (e.g. *S. coffeanum* and *S. filiforme* isolated from *Coffea arabica*^6^ and *Citrullus lanatus*^7^), from decaying wood or rock (*S. calcicola*^8^ and *S. chinense*^9^) or from multiple sources. *Simplicillium obclavatum* was isolated from air, soil, bark, human nail, and seawater^1,10^, whereas *S. aogashimaense* was isolated from soil, seawater, and as symbiotic fungi from *Nilaparvata lugens* Stål^5,11,12^. *Simplicillium lamellicola* was isolated as endophytic, entomopathogenic, and mycoparasitic fungi^1,13–15^. *Simplicillium lanosoniveum* was isolated as cyanobacterium-symbiotic, endophytic, entomopathogenic, and mycoparasitism fungi^16–19^. Among those *Simplicillium* species, six species viz. *S. cicadellidae*, *S. formicae*, *S. formicidae*, *S. lamellicola*, *S. lanosoniveum* and *S. lepidopterorum*, were found associated with insects.

In the present study, four novel insect-associated species viz. *Simplicillium coccinellidae*, *S. hymenopterorum*, *S. neolepidopterorum* and *S. scarabaeoidea*, were introduced based on morphological comparison and molecular phylogenetic analyses, and this may contribute to the control of insect pest and the discovery of useful novel compounds.

## Result

### Phylogenetic analyses

In the phylogenetic tree, *Purpureocillium lilacinum* (Thom) Luangsa-ard, Houbraken, Hywel-Jones & Samson (CBS 284.36 and CBS 431.87) and *Pochonia chlamydosporia* (Goddard) Zare & W. Gams (CBS 103.65) were used as the outgroup in analysis 1 and analysis 2, respectively. The concatenated sequences of analysis 1 and analysis 2 included 46 and 22 taxa, and consisted of 1,729 (*LSU*: 497, *RPB1*: 550 and *TEF*: 682) and 1,904 (*SSU*: 845, *ITS*: 541 and *LSU*: 518) characters with gaps, respectively.

**Figure 1:**
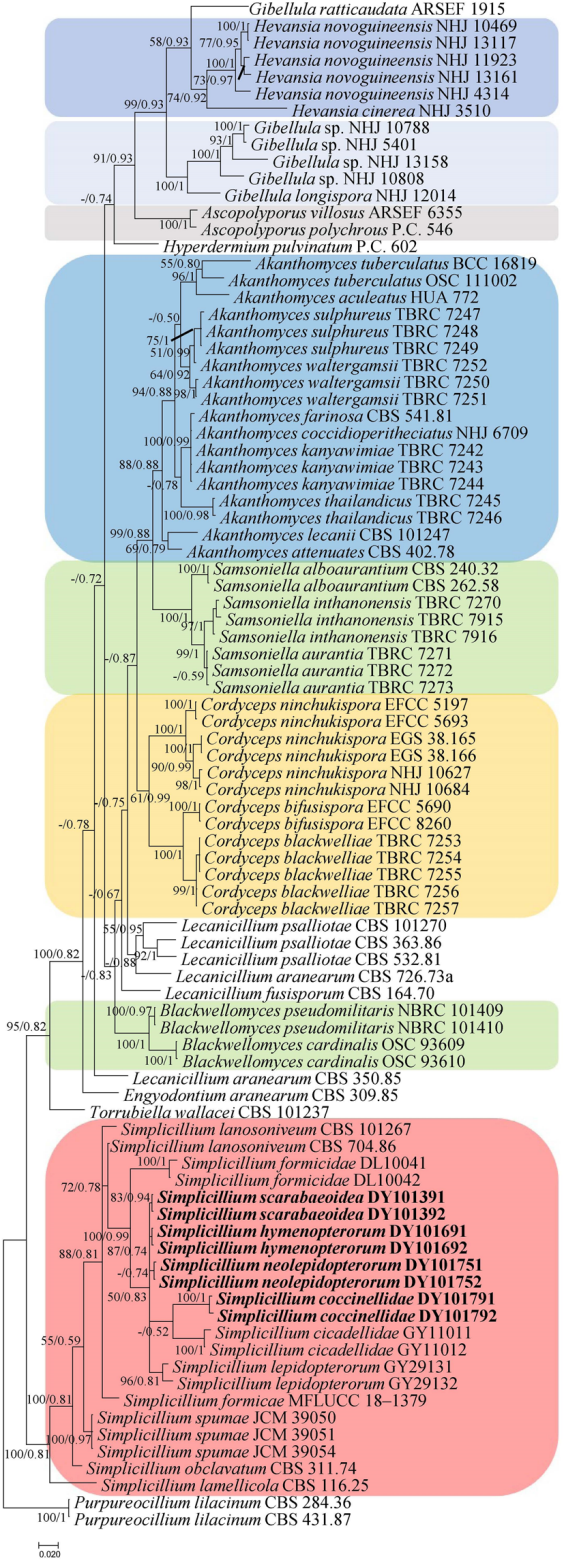
Phylogenetic relationships among the genus *Simplicillium* and closely-related species in Cordycipitaceae based on multigene dataset (*LSU*, *RPB1* and *TEF*). Statistical support values (≥ 70%/0.7) are shown at the nodes for ML bootstrap support/BI posterior probabilities.

Analysis 1: The P-value of PAUP4.0b10 using the command “hompart” is 0.01, and indicated the dataset *LSU* + *RPB1* + *TEF* is not suitable for the combined analysis. The selected model for *LSU*, *RPB1* and *TEF* were SYM + G4, SYM + G4 and GTR + F + I + G4, respectively. The final value of the highest scoring tree was –17,856.725706, which was obtained from the ML analysis of the dataset (*LSU* + *RPB1* + *TEF*). The parameters of GTR model to analysis of the dataset were estimated base frequencies; A = 0.235757, C = 0.286704, G = 0.270379, T = 0.207160; substitution rates AC = 0.874437, AG = 2.344268, AT = 0.877112, CG = 0.872563, CT = 6.144163, GT = 1.000000; gamma distribution shape parameter α = 0.441982. In the phylogenetic tree (Fig. 1), both analyses of ML and BI trees were largely congruent, and strongly supported in most branches. All *Simplicillium* species were nested in an independent clade, which was the earliest diverging lineage in Cordycipitaceae. The four new species, *S. coccinellidae*, *S. hymenopterorum*, *S. neolepidopterorum* and *S. scarabaeoidea* were both formed an independent branch and clustered with *S. cicadellidae*, *S. formicidae* and *S. lepidopterorum* in a subclade.

**Figure 2:**
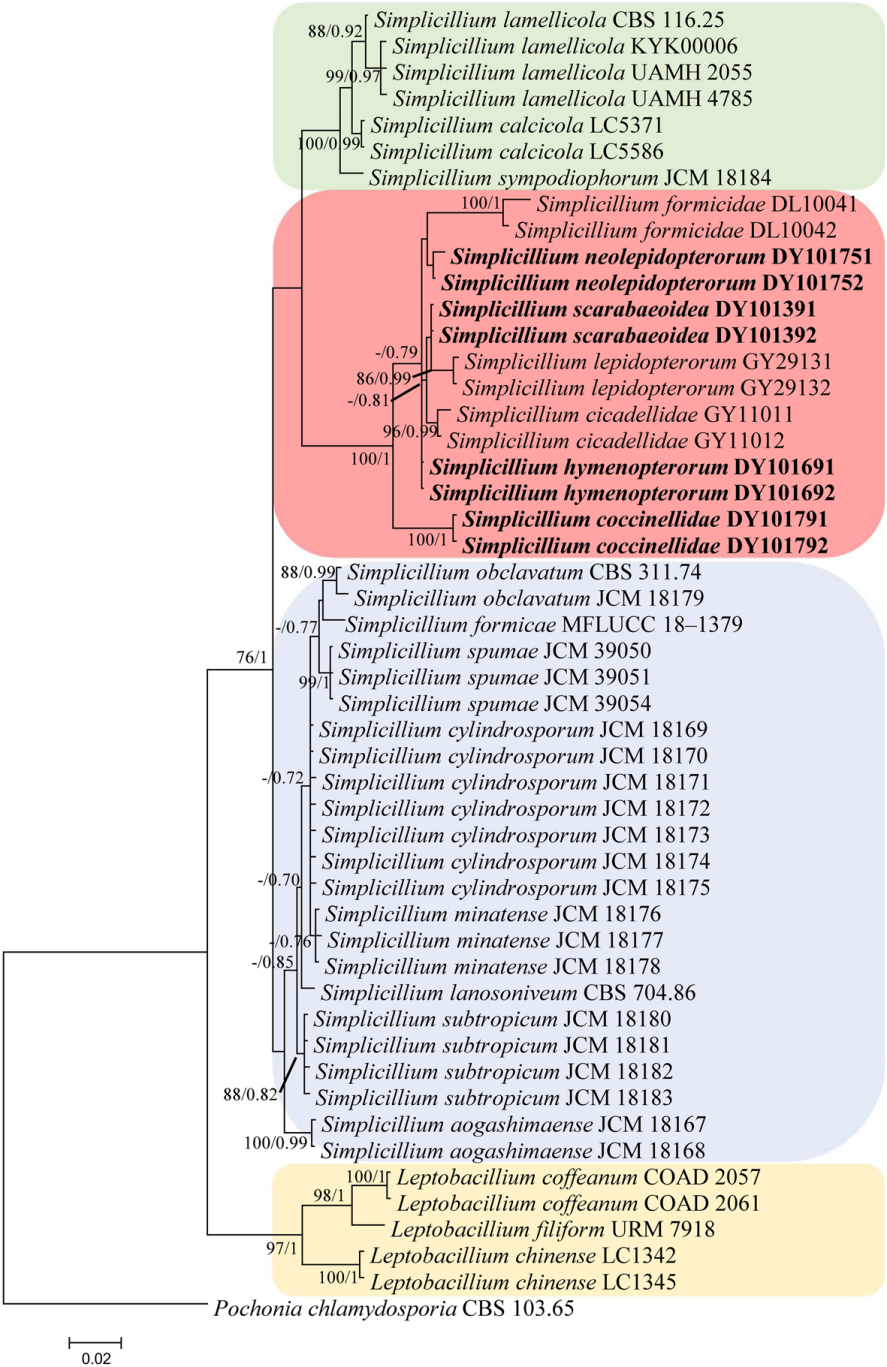
Phylogenetic relationships among the new taxa, other *Simplicillium* species and closely-related species by *SSU* + *ITS* + *LSU* sequences. Statistical support values (≥ 70%/0.7) are shown at the nodes for ML bootstrap support/BI posterior probabilities.

Analysis 2: The P-value of PAUP4.0b10 using the command “hompart” is 0.99, and indicated the dataset *SSU* + *ITS* + *LSU* is suitable for the combined analysis. The selected model was JC for *SSU* and K2P + G4 for *ITS* + *LSU*. The final value of the highest scoring tree was –6,637.139922, which was obtained from the ML analysis of the dataset (*SSU* + *ITS* + *LSU*). The parameters of GTR model to analysis of the dataset were estimated base frequencies; A = 0.251177, C = 0.239762, G = 0.263036, T = 0.246025; substitution rates AC = 1.301732, AG = 2.440073, AT = 0.844382, CG = 1.306407, CT = 3.262235, GT = 1.000000; gamma distribution shape parameter α = 0.552466. In the phylogenetic tree (Fig. 2), both analyses of ML and BI trees were largely congruent, and strongly supported in most branches. Four well-supported clades representing four new novel species *S. coccinellidae*, *S. hymenopterorum*, *S. neolepidopterorum* and *S. scarabaeoidea* were obtained. These new species clustered with *S. cicadellidae*, *S. formicidae* and *S. lepidopterorum* in a well-supported subclade within the *Simplicillium* lineage. *S. coccinellidae* and *S. hymenopterorum* were both nested in an independent clade. *S. neolepidopterorum* and *S. scarabaeoidea* have a close relationship with *S. formicidae* and *S. lepidopterorum*, respectively.

## Taxonomy

**Figure 3:**
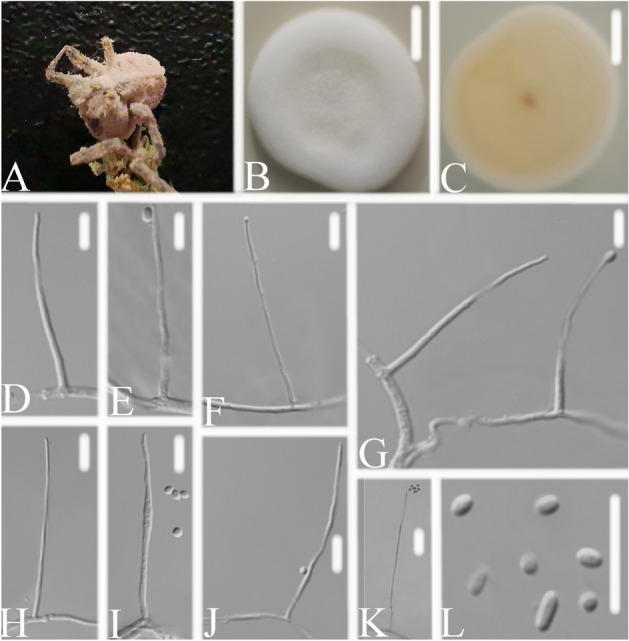
*Simplicillium coccinellidae* **(A)** infected ladybug (Coccinellidae) **(B,C)** culture plate, showing the front **(B)** and the reverse **(C)** of the colony, cultured on PDA medium **(D–K)** phialides solitary, conidia adhering ellipsoidal slimy head and conidia L conidia. Scale bars: 10 mm **(B,C)**, 10 μm **(D–L)**.

*Simplicillium coccinellidae* W.H. Chen, Y.F. Han, Z.Q. Liang sp. nov. (Fig. 3).

*MycoBank No.*: MB 835583.

*Etymology*: referring to its insect host, family Coccinellidae.

*Description*: The colonies were moderate-growing on PDA medium, reaching a diameter of 31–36 mm, in 14 days at 25 °C, convex, with white velutinate aerial mycelium, reverse yellowish to pale brown, especially in the middle, margin entire, soluble pigment not produced. *Vegetative hyphae* branched, hyaline, smooth-walled, septate, 1.1–1.9 μm wide. *Phialides* produced on aerial hyphae, always solitary, aseptate, hyaline, smooth-walled, relatively slender, and tapering toward the tip, 24.9–62.1 × 1.0–1.5 μm. *Conidia* in small subglobose slimy heads at the apex of the phialides, hyaline, cylindrical, ellipsoidal to globose, aseptate, smooth-walled, 1-celled, 2.0–3.4 × 1.6–2.0 μm, Octahedral crystals absent.

*Material examined*: CHINA, Guizhou, Guiyang, Duyun City (26°21′27.96″ N, 107°22′48.22″ E). On dead sacrab (Coccinellidae), 1 October 2019, Wanhao Chen, DY10179 (GZAC DY10179, holotype), was deposited at the Institute of Fungus Resources, Guizhou University (formally Herbarium of Guizhou Agricultural College; code, GZAC), Guiyang City, Guizhou, China; ex-type living cultures, DY101791, DY101792. Sequences from isolated strain DY101791 has been deposited in GenBank with accession numbers: *ITS* = MT453861, *SSU* = MT453863, *LSU* = MT453862 and *TEF* = MT471341.

*Know distribution*: China, Guizhou Province, Duyun City (26°21′27.96″ N, 107°22′48.22″ E).

*Notes*: *S. coccinellidae* share similar conidial and phialide morphologies with the related species (Table 1). However, the pairwise dissimilarities of *ITS* sequences show 30, 127, 31, 29, 45, 33 bp difference within 584 bp between *S. coccinellidae* and *S. cicadellidae*, *S. formicidae*, *S. lepodopterorum*, *S. hymenopterorum*, *S. neolepidopterorum*, *S. scarabaeoidea* respectively. Jeewon & Hyde^39^ recommended that a minimum of > 1.5% nucleotide differences in the ITS regions may be indicative of a new species. Besides, based on the analysis of the combined dataset *LSU* + *RPB1* + *TEF* and *SSU* + *ITS* + *LSU*, *S. coccinellidae* was nested in a separate group in both phylogenetic trees. Thus, the molecular phylogenetic results supported that *S. coccinellidae* was a new species in the genus *Simplicillium*.

**Table 1:** Morphological comparison of four new species with other *Simplicillium* species.

Species	Morphological characteristics		
	Phialide (Conidiogenous cell) (μm)	Conidia (μm)	Conidia mass
*S. cicadellidae*	12.9–18.3 × 0.8–1.1	Ellipsoidal, 1.8–2.8 × 1.4–1.8	Ellipsoidal heads
*S. formicidae*	51–70.1 × 0.7–0.9	Filiform to fusoid, 3.9–7.9 × 0.8–1.3	Globose heads
*S. lepodopterorum*	15.3–26.2 × 0.7–1.4	Ellipsoidal, 1.6–2.4 × 1.4–1.7	Globose heads
***S. coccinellidae***	**24.9–62.1 × 1.0–1.5**	**Cylindrical, ellipsoidal to globose, 2.0–3.4 × 1.6–2.0**	**Subglobose heads**
***S. hymenopterorum***	**19.3–46.2 × 1.1–2.3**	**Cylindrical to subellipsoidal, 2.1–2.8 × 1.3–1.9**	**Subglobose heads**
***S. neolepidopterorum***	**34.1–44.3 × 1.0–1.7**	**Ellipsoidal to cylindrical, 2.5–3.8 × 1.5–2.1**	
***S. scarabaeoidea***	**18.5–63.4 × 1.1–1.4**	**Ellipsoidal, 1.9–2.9 × 1.4–2.0**	**Globose heads**

Sequences generated in this study are shown in bold.

**Figure 4:**
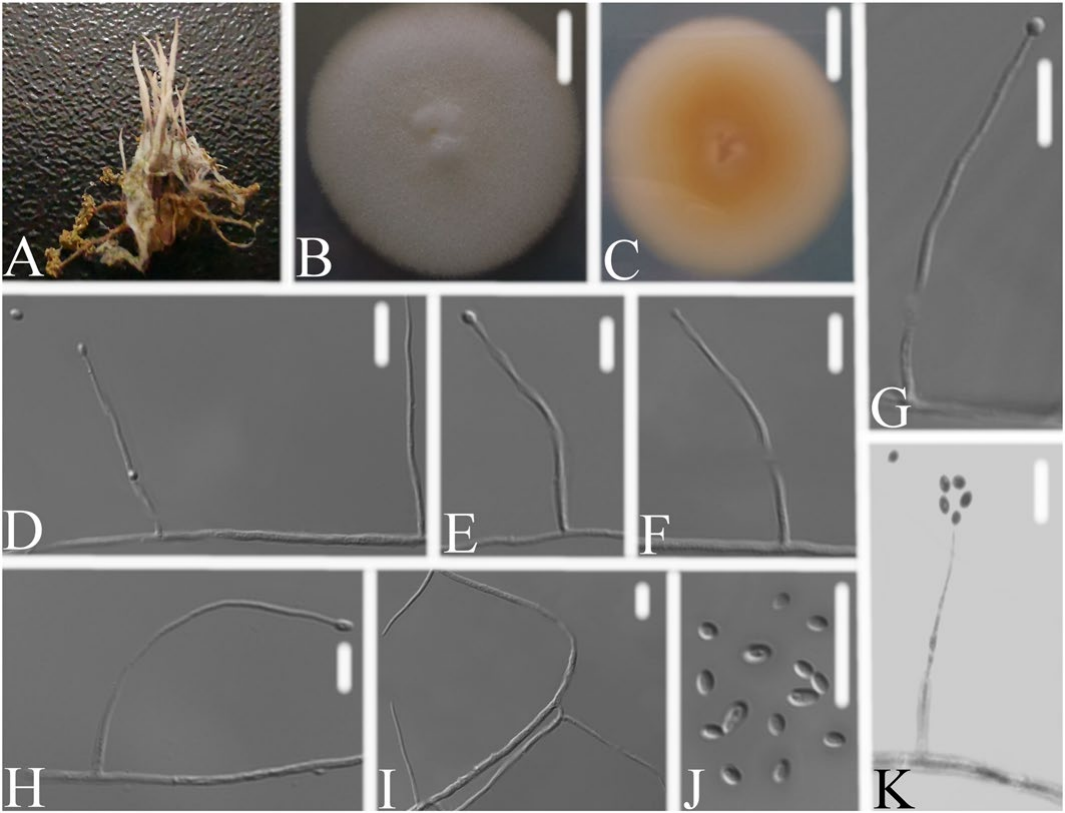
*Simplicillium hymenopterorum* **(A)** infected ant (Hymenoptera) **(B,C)** culture plate, showing the front **(B)** and the reverse **(C)** of the colony, cultured on PDA medium **(D–I)**, **(K)** phialides solitary, conidia adhering ellipsoidal slimy head and conidia **(J)** conidia. Scale bars: 10 mm **(B,C)**, 10 μm **(D–K)**.

*Simplicillium hymenopterorum* W.H. Chen, Y.F. Han, Z.Q. Liang sp. nov. (Fig. 4).

*MycoBank No.* : MB 835581.

*Etymology*: referring to its insect host, order Hymenoptera.

*Description*: The colonies were rapid-growing on PDA medium, reaching a diameter of 40–42 mm, in 14 days at 25 ℃, convex, with white velutinate aerial mycelium, reverse pale yellow, especially in the middle, margin entire, soluble pigment not produced. *Phialides* produced on prostrate aerial hyphae, mainly solitary, aseptate, hyaline, smooth-walled, relatively slender, and tapering toward the tip, 19.3–46.2 × 1.1–2.3 μm. *Conidia* in small subglobose heads at the apex of the phialides, hyaline, cylindrical to subellipsoidal, aseptate, smooth-walled, 1-celled, 2.1–2.8 × 1.3–1.9 μm, Octahedral crystals absent.

*Material examined*: CHINA, Guizhou, Guiyang, Duyun City (26°21′27.96″ N, 107°22′48.22″ E). On dead ant (Hymenoptera), 1 October 2019, Wanhao Chen, DY10169 (GZAC DY10169, holotype), was deposited at the Institute of Fungus Resources, Guizhou University (formally Herbarium of Guizhou Agricultural College; code, GZAC), Guiyang City, Guizhou, China; ex-type living cultures, DY101691, DY101692. Sequences from isolated strain DY101691 has been deposited in GenBank with accession numbers: *ITS* = MT453848, *SSU* = MT453849, *LSU* = MT453850, *RPB1* = MT471344 and *TEF* = MT471337.

*Notes*: Based on the analysis of the combined dataset *LSU* + *RPB1* + *TEF* and *SSU* + *ITS* + *LSU*, *S. hymenopterorum* was nested in a separate group in two phylogenetic trees. The pairwise dissimilarities of *ITS* sequences show 105, 24, 31, 17 bp difference within 582 bp between *S. hymenopterorum* and *S. formicidae*, *S. lepodopterorum*, *S. coccinellidae*, *S. neolepidopterorum*, respectively. The pairwise dissimilarities of *RPB1* sequences show 25, 16 bp difference within 737 bp between *S. hymenopterorum* and *S. cicadellidae*, *S. scarabaeoidea* respectively. When compared with the typical characteristics of *S. cicadellidae* and *S. scarabaeoidea* (Table 1), *S. hymenopterorum* could be easily distinguished from *S. cicadellidae* and *S. scarabaeoidea* by having subglobose slimy heads of conidia, cylindrical to subellipsoidal conidia, 2.1–2.8 × 1.3–1.9 μm and phialides, 19.3–46.2 × 1.1–2.3 μm. Thus, morphologically based conclusion supported the molecular phylogenetic results that *S. hymenopterorum* was a new species in the genus *Simplicillium*.

**Figure 5:**
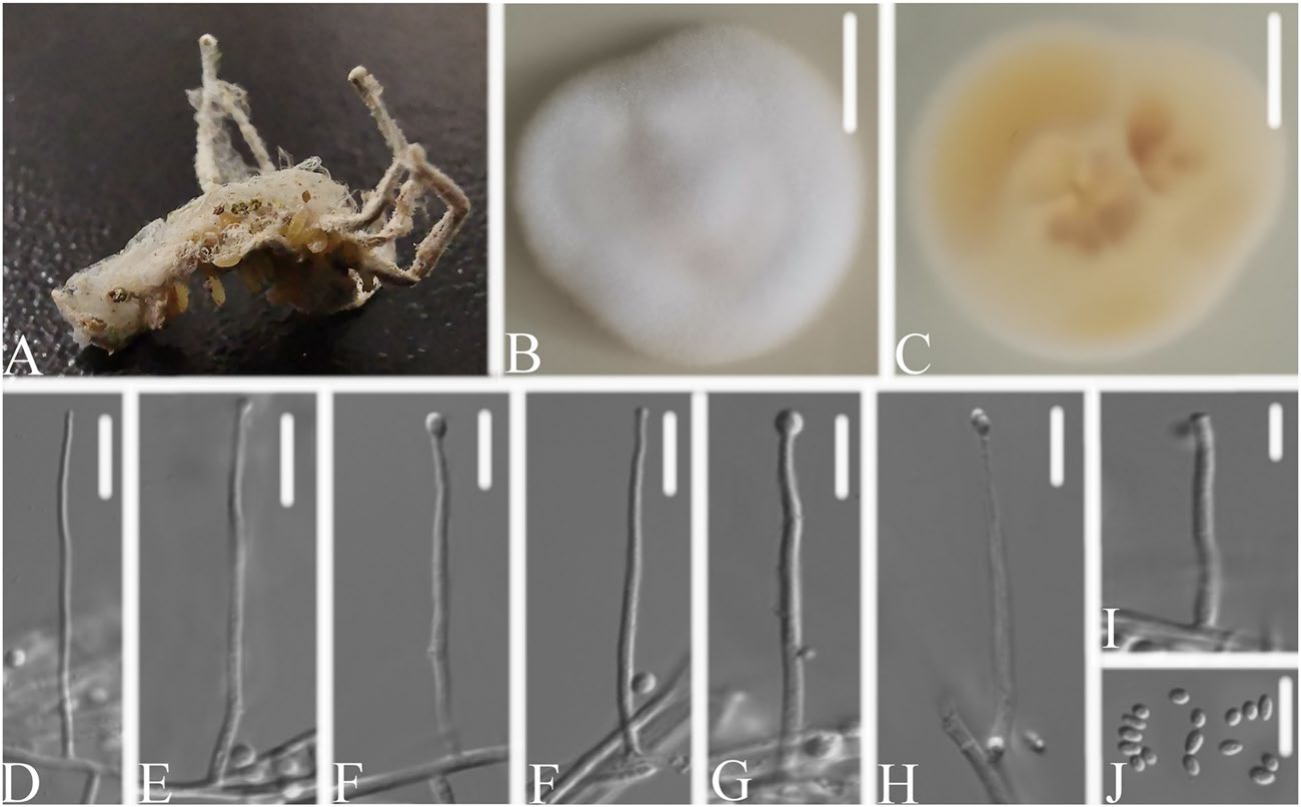
*Simplicillium neolepidopterorum* **(A)** infected moth (Lepidoptera) **(B,C)** culture plate, showing the front **(B)** and the reverse **(C)** of the colony, cultured on PDA medium **(D–I)** phialides solitary, conidia adhering ellipsoidal slimy head and conidia **(J)** conidia. Scale bars: 10 mm **(B,C)**, 10 μm **(D–J)**.

*Simplicillium neolepidopterorum* W.H. Chen,Y.F. Han, Z.Q. Liang sp. nov. (Fig. 5).

*MycoBank No.* : MB 835582.

*Etymology*: referring to its insect host, order Lepidoptera.

*Description*: *Insect host* was completely covered by white to yellowish, loosely mycelium. *Conidiophore* mononematous. The colonies were slow-growing on PDA medium, reaching a diameter of 28–31 mm, in 14 days at 25 ℃, convex, with white velutinate aerial mycelium, reverse yellowish to pale brown, especially in the middle, margin entire, soluble pigment not produced. *Vegetative hyphae* branched, hyaline, septate, smooth-walled, 1.3–1.4 μm wide. *Phialides* produced on aerial hyphae, always solitary and rather long and narrow, aseptate, hyaline, smooth-walled, relatively slender, and tapering toward the tip, 34.1–44.3 × 1.0–1.7 μm. *Conidia* solitary, occasionally in short imbricate chains, hyaline, ellipsoidal to cylindrical, aseptate, smooth-walled, 1-celled, 2.5–3.8 × 1.5–2.1 μm, Octahedral crystals absent.

*Material examined*: CHINA, Guizhou, Guiyang, Duyun City (26°21′27.96″ N, 107°22′48.22″ E). On dead insect (Lepidoptera), 1 October 2019, Wanhao Chen, DY10175 (GZAC DY10175, holotype), was deposited at the Institute of Fungus Resources, Guizhou University (formally Herbarium of Guizhou Agricultural College; code, GZAC), Guiyang City, Guizhou, China; ex-type living cultures, DY101751, DY101752. Sequences from isolated strain DY101751 has been deposited in GenBank with accession numbers: *ITS* = MT453854, *SSU* = MT453856, *LSU* = MT453855 and *TEF* = MT471339.

*Notes*: Based on the analysis of the combined dataset *SSU* + *ITS* + *LSU*, *S. neolepidopterorum* is phylogenetically close to *S. formicidae*. Besides, the pairwise dissimilarities of *ITS* sequences show 153 bp difference within 580 bp between S. *neolepidopterorum* and *S. formicidae*. When compared with the typical characteristics of *S. formicidae* (Table 1), *S. neolepidopterorum* could easily distinguished from *S. formicidae* by having solitary conidia, occasionally in short imbricate chains, and ellipsoidal to cylindrical conidia. Thus, molecular phylogenetic results and morphologically based conclusion were supported *S. neolepidopterorum* was a new species in the genus *Simplicillium*.

**Figure 6:**
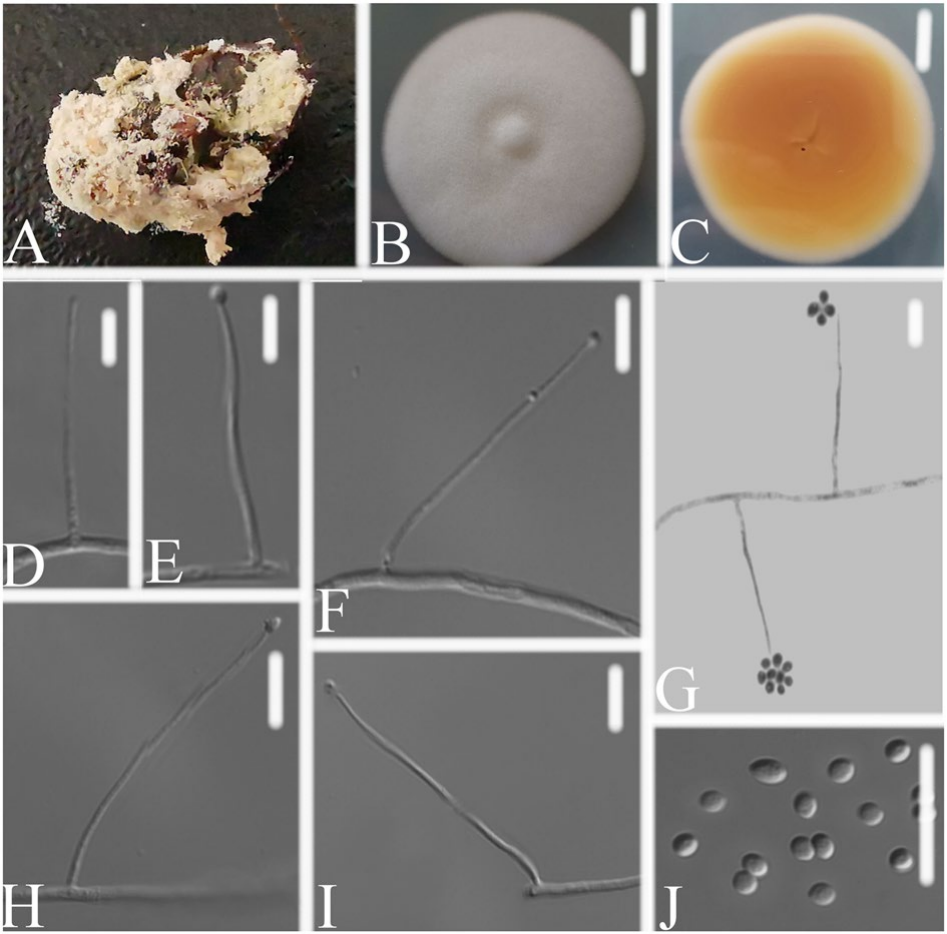
*Simplicillium scarabaeoidea* **(A)** infected scarab (Scarabaeoidea) **(B,C)** culture plate, showing the front **(B)** and the reverse **(C)** of the colony, cultured on PDA medium **(D–I)** phialides solitary, conidia adhering ellipsoidal slimy head and conidia **(J)** conidia. Scale bars: 10 mm **(B,C)**, 10 μm **(D–J)**.

*Simplicillium scarabaeoidea *W.H. Chen, Y.F. Han, Z.Q. Liang sp. nov. (Fig. 6).

*MycoBank No.* : MB 835580.

*Etymology*: referring to its insect host, family Scarabaeoidea.

*Distribution*: *Insect host* was completely covered by white, yellowish to pinkish, densely mycelium. *Conidiophore* mononematous. The colonies were rapid-growing on PDA medium, reaching a diameter of 44–47 mm, in 14 days at 25 ℃, convex, with white velutinate aerial mycelium; reverse pale yellow, margin entire, soluble pigment not produced. *Phialides* produced on prostrate aerial hyphae, mainly solitary, aseptate, hyaline, smooth-walled, relatively slender, and tapering toward the tip, 18.5–63.4 × 1.1–1.4 μm. *Conidia* in small globose heads at the apex of the phialides, hyaline, ellipsoidal, aseptate, smooth-walled, 1-celled, 1.9–2.9 × 1.4–2.0 μm. Octahedral crystals absent.

*Material examined*: CHINA, Guizhou, Guiyang, Duyun City (26°21′27.96″ N, 107°22′48.22″ E). On dead insect (Lepidoptera), 1 October 2019, Wanhao Chen, DY10139 (GZAC DY10139, holotype), was deposited at the Institute of Fungus Resources, Guizhou University (formally Herbarium of Guizhou Agricultural College; code, GZAC), Guiyang City, Guizhou, China; ex-type living cultures, DY101391, DY101392. Sequences from isolated strain DY101391 has been deposited in GenBank with accession numbers: *ITS* = MT453842, *SSU* = MT453843, *LSU* = MT453844, *RPB1* = MT471343 and *TEF* = MT471335.

*Notes*: Based on the analysis of the combined dataset *SSU* + *ITS* + *LSU*, *S. scarabaeoidea* is phylogenetically close to *S. lepodopterorum*. However, the pairwise dissimilarities of *RPB1* sequences show 31 bp difference within 760 bp between *S. scarabaeoidea* and *S. lepodopterorum*. When comparing with the typical characteristics of *S. lepodopterorum* (Table 1), *S. scarabaeoidea* could be easily distinguished from *S. lepodopterorum* by having longer phialides and larger conidia. Thus, molecular phylogenetic results and morphologically based conclusion were supported *S. scarabaeoidea* was a new species in the genus *Simplicillium*.

*Leptobacillium filiform* (R.M.F. Silva, R.J.V. Oliveira, Souza-Motta, J.L. Bezerra & G.A. Silva) W.H. Chen, Y.F. Han J.D. Liang & Z.Q. Liang, *comb. nov.*

Mycobank No.: MB839923.

Basionym: *Simplicillium filiform* R.M.F. Silva, R.J.V. Oliveira, Souza-Motta, J.L. Bezerra & G.A. Silva, *Persoonia* 41: 403 (2018).

*Notes*: Okane et al.^3^ transferred *Simplicillium chinense* and *S. coffeanum* to the genus *Leptobacillium*. In the present study, *S. chinense*, *S. coffeanum* and *S. filiform* were clustered into an independent clade (Fig. 2), and supported by Crous et al.^7^, Chen et al.^20^ and Wei et al.^28^. Thus, *L. filiform* is proposed as a new combination.

## Discussion

Sung et al.^40^ refined the classification of *Cordyceps* and the Clavicipitaceae; the genus *Simplicillium* thus belongs to the Cordycipitaceae sensu stricto. The result of phylogenetic analysis of the combined dataset (*SSU*, *LSU*, *RPB1*, *RPB2* and *TEF*) showed that *Simplicillium* species were all clustered in an independent group and as the most ancient lineage in the phylogenetic tree^41^. In this study, all *Simplicillium* species were also clustered into a clade at the end of the tree (Fig. 1) based on the analysis of the concentrated dataset (*LSU*, *RPB1* and *TEF*). The four newly identified species, *S. scarabaeoidea*, *S. hymenopterorum*, *S. neolepidopterorum* and *S. coccinellidae*, were all clustered in a separate subclade. Liu & Cai^9^ reported a new species based on the morphological comparison and phylogenetic analysis of *ITS* and *LSU* sequences, which was the earliest application for the identification of *Simplicillium* species. Kondo et al.^29^ added the loci *SSU* in the analysis of *Simplicillum* species. Thus, three loci (*ITS*, *LSU* and *SSU*) were applied in the analysis of the relationship among *Simplicillium* species in this study.

The nutritional mode from plant to animals and fungi is the evolutionary characteristics of Hypocreales^42^. Plants associated fungi, which including living plants and plant residues were the common ancestor in the families Hypocreaceae and Clavicipitaceae^41^. The animal pathogenic fungi are likely inherited from the plant associated fungi by a series of interkingdom host jumps^42^. In the phylogenetic tree of analysis 2 (Fig. 2), *S. chinense*, *S. filiforme* and *S. coffeanum* were nested in a clade and at the end of the tree. The substrates of *S. chinense*, *S. coffeanum* and *S. filiforme* were decaying wood, branches of *Coffea arabica* and leaves of *Citrullus lanatus*^6,7,9^. All of them were belongs to plants associated fungi, and might reflect the initial state of *Simplicillium* species, which then underwent a host jump or transferred their nutritional preference. *Simplicillium* species have rich diversity in substrates and life modes, such as soil, seawater, air, and isolated as symbiotic, endophytic, entomopathogenic and mycoparasitic fungi. *Simplicillium* species associated with predatory insects or animals, like spiders, will likely soon be reported. Thus, the genus *Simplicillium* will completely fit with the nutritional model of Hypocreales fungi and could be used as a model to study the evolutionary relationship.

Numerous new secondary metabolites were found from *Simplicillium* species, such as alkaloids^43^, diketopiperazine^44^ and anthraquinones^45^, especially aogacillin A, B and Simpotentin, which have antibacterial and antifungal activities and shown great potential applications in medicine^46,47^. In addition, some *Simplicillium* species were isolated as symbiotic, entomopathogenic and mycoparasitic fungi, and could be used to biocontrol of insect pest, nematode and microbial diseases^48–50^. Thus, it is expected that useful novel compounds will be discovered from the newly-reported *Simplicillium* species described here and be a natural resource for the application in biocontrol, medicine and health.

## Materials and methods

### Specimen collection and identification

Four infected insect specimens (DY10139, DY10169, DY10175 and DY10179) were collected from Duyun City (26°21′24.71″ N, 107°22′48.22″ E), Guizhou Province, on 1 October, 2019. Isolation of strains was conducted as described by Chen et al.^20^. Fungal colonies emerging from specimens were isolated and cultured at 25 °C for 14 days under 12 h light/12 h dark conditions following protocols described by Zou et al.^21^. Accordingly, strains were obtained. The specimens and the isolated strains were deposited in the Institute of Fungus Resources, Guizhou University (formally Herbarium of Guizhou Agricultural College; code, GZAC), Guiyang City, Guizhou, China.

Macroscopic and microscopic morphological characteristics of the fungi were examined and the growth rates were determined from PDA cultures incubated at 25 °C for 14 days. Hyphae and conidiogenous structures were mounted in lactophenol cotton blue or 20% lactate solution and observed with an optical microscope (OM, DM4 B, Leica, Germany).

DNA extraction, polymerase chain reaction amplification and nucleotide sequencing.

DNA extraction was carried out by Fungal genomic DNA Extraction Kit (DP2033, BioTeke Corporation) in accordance with Liang et al.^22^. The extracted DNA was stored at − 20 °C. The amplification of internal transcribed spacer (*ITS*) region, small subunit ribosomal RNA (*SSU*), large subunit ribosomal RNA (*LSU*) gene, RNA polymerase II largest subunit 1 (*RPB1*) and translation elongation factor 1 alpha (*TEF*) were amplified by PCR as described by White et al.^23^, Rakotonirainy et al.^24^, Castlebury et al.^25^ and van den Brink et al.^26^, respectively. PCR products were purified and sequenced at Sangon Biotech (Shanghai) Co. The generated sequences were submitted to GenBank.

### Sequence alignment and phylogenetic analyses

Lasergene software (version 6.0, DNASTAR) was applied for the assembling and editing of DNA sequence in this study. The *ITS*, *LSU*, *SSU*, *RPB1* and *TEF* sequences were downloaded from GenBank, based on Nonaka et al.^5^, Zhang et al.^8^, Gomes et al.^6^, Crous et al.^7^, Mongkolsamrit et al.^27^, Chen et al.^20^, Wei et al.^28^, Kondo et al.^29^ and others selected on the basis of BLAST algorithm-based searches in GenBank (Table 2). The Multiple datasets of *ITS*, *LSU*, *SSU*, *RPB1* and *TEF* were aligned and edited by MAFFT v7.037b^30^ and MEGA6^31^. Assembling of the combined datasets (*LSU* + *RPB1* + *TEF* and *SSU* + *ITS* + *LSU*) were performed by SequenceMatrix v.1.7.8^32^. The partition homogeneity test was conducted in PAUP4.0b10^33^ by using the command “hompart”.

**Table 2:** Taxa included in the phylogenetic analyses.

Species	Strain no.	GenBank accession no.				
		ITS	SSU	LSU	RPB1	TEF
*Akanthomyces aculeatus*	HUA 772			KC519370		KC519366
*A. attenuatus*	CBS 402.78			AF339565	EF468888	EF468782
*A. coccidioperitheciatus*	NHJ 6709			EU369042	EU369067	EU369025
*A. farinosa*	CBS 541.81					JQ425686
*A. kanyawimiae*	TBRC 7242			MF140718	MF140784	MF140838
*A. kanyawimiae*	TBRC 7243			MF140717	MF140783	MF140837
*A. kanyawimiae*	TBRC 7244			MF140716		MF140836
*A. lecanii*	CBS 101247			AF339555	DQ522407	DQ522359
*A. sulphureus*	TBRC 7247			MF140720		MF140841
*A. sulphureus*	TBRC 7248			MF140722	MF140787	MF140843
*A. sulphureus*	TBRC 7249			MF140721	MF140786	MF140842
*A. thailandicus*	TBRC 7245					MF140839
*A. thailandicus*	TBRC 7246			MF140719		MF140840
*A. tuberculatus*	BCC 16819			GQ249987		GQ250037
*A. tuberculatus*	OSC 111002			DQ518767	DQ522384	DQ522338
*A. waltergamsii*	TBRC 7250			MF140715		MF140835
*A. waltergamsii*	TBRC 7251			MF140713	MF140781	MF140833
*A. waltergamsii*	TBRC 7252			MF140714	MF140782	MF140834
*Ascopolyporus polychrous*	P.C. 546			DQ118737	DQ127236	DQ118745
*A. villosus*	ARSEF 6355			AY886544	DQ127241	DQ118750
*Blackwellomyces cardinalis*	OSC 93609			AY184962	DQ522370	DQ522325
*B. cardinalis*	OSC 93610			AY184963	EF469088	EF469059
*B. pseudomilitaris*	NBRC 101409			JN941393	JN992482	
*B. pseudomilitaris*	NBRC 101410			JN941394	JN992481	
*Cordyceps bifusispora*	EFCC 5690			EF468806	EF468854	EF468746
*C. bifusispora*	EFCC 8260			EF468807	EF468855	EF468747
*C. blackwelliae*	TBRC 7253			MF140705	MF140774	MF140825
*C. blackwelliae*	TBRC 7254			MF140704	MF140773	MF140824
*C. blackwelliae*	TBRC 7255			MF140703	MF140772	MF140823
*C. blackwelliae*	TBRC 7256			MF140702	MF140771	MF140822
*C. blackwelliae*	TBRC 7257			MF140701	MF140770	MF140821
*C. ninchukispora*	EFCC 5197			EF468820	EF468868	EF468760
*C. ninchukispora*	EFCC 5693			EF468821	EF468869	EF468762
*C. ninchukispora*	EGS 38.165			EF468846	EF468900	EF468795
*C. ninchukispora*	EGS 38.166			EF468847	EF468901	EF468794
*C. ninchukispora*	NHJ 10627			EF468822	EF468870	EF468763
*C. ninchukispora*	NHJ 10684			EF468823	EF468871	EF468761
*Engyodontium aranearum*	CBS 309.85			AF339526	DQ522387	DQ522341
*Gibellula longispora*	NHJ 12014				EU369055	EU369017
*G. pulchra*	NHJ 10808			EU369035	EU369056	EU369018
*G. ratticaudata*	ARSEF 1915			DQ518777	DQ522408	DQ522360
*Gibellula sp.*	NHJ 5401				EU369059	
*Gibellula sp.*	NHJ 10788			EU369036	EU369058	EU369019
*Gibellula sp.*	NHJ 13158			EU369037	EU369057	EU369020
*Hevansia arachnophila*	NHJ 10469			EU369031	EU369047	EU369008
*H. cinerea*	NHJ 3510				EU369048	EU369009
*H. novoguineensis*	NHJ 4314				EU369051	EU369012
*H. novoguineensis*	NHJ 11923			EU369032	EU369052	EU369013
*H. novoguineensis*	NHJ 13117				EU369049	EU369010
*H. novoguineensis*	NHJ 13161				EU369050	EU369011
*Hyperdermium pulvinatum*	P.C. 602			AF242353	DQ127237	DQ118746
*Lecanicillium aranearum*	CBS 726.73a			AF339537	EF468887	EF468781
*L. antillanum*	CBS 350.85			AF339536	DQ522396	DQ522350
*L. fusisporum*	CBS 164.70			AF339549	EF468889	EF468783
*L. psalliotae*	CBS 363.86			AF339559	EF468890	EF468784
*L. psalliotae*	CBS 532.81			AF339560	EF469096	EF469067
*L. psalliotae*	CBS 101270			EF469081	EF469095	EF469066
*Pochonia chlamydosporia*	CBS 103.65	MH858504				
*Purpureocillium lilacinum*	CBS 284.36			FR775484	EF468898	EF468792
*P. lilacinum*	CBS 431.87			EF468844	EF468897	EF468791
*Samsoniella alboaurantium*	CBS 240.32			JF415979	JN049895	JF416019
*S. alboaurantium*	CBS 262.58			MG665232		JQ425685
*S. aurantia*	TBRC 7271			MF140728	MF140791	MF140846
*S. aurantia*	TBRC 7272			MF140727	MF140817	MF140845
*S. aurantia*	TBRC 7273			MF140726		MF140844
*S. inthanonensis*	TBRC 7915			MF140725	MF140790	MF140849
*S. inthanonensis*	TBRC 7916			MF140724	MF140789	MF140848
*S. inthanonensis*	TBRC 7270			MF140723	MF140788	MF140847
*Simplicillium aogashimaense*	JCM 18167	AB604002				
*S. aogashimaense*	JCM 18168	AB604004				
*S. calcicola*	LC5371	KU746705		KU74675		
*S. calcicola*	LC5586	KU746706		KU746752		
*S. chinense*	LC1342	JQ410323		JQ410321		
*S. chinense*	LC1345	NR 155782		JQ410322		
*S. cicadellidae*	GY11011	MN006243			MN022271	MN022263
*S. cicadellidae*	GY11012	MN006244			MN022272	MN022264
*S. coffeanum*	COAD 2057	MF066034		MF066032		
*S. coffeanum*	COAD 2061	MF066035		MF066033		
*S. cylindrosporum*	JCM 18169	AB603989				
*S. cylindrosporum*	JCM 18170	AB603994				
*S. cylindrosporum*	JCM 18171	AB603997				
*S. cylindrosporum*	JCM 18172	AB603998				
*S. cylindrosporum*	JCM 18173	AB603999				
*S. cylindrosporum*	JCM 18174	AB604005				
*S. cylindrosporum*	JCM 18175	AB604006				
*S. filiforme*	URM 7918	MH979338		MH979399		
*S. formicae*	MFLUCC 18–1379	MK766511	MK765046	MK766512	MK882623	MK926451
*S. formicidae*	DL10041	MN006241			MN022269	
*S. formicidae*	DL10042	MN006242			MN022270	
*S. lamellicola*	CBS 116.25	AJ292393		AF339552	DQ522404	DQ522356
*S. lamellicola*	KYK00006	AB378533				
*S. lamellicola*	UAMH 2055	AF108471				
*S. lamellicola*	UAMH 4785	AF108480				
*S. lanosoniveum*	CBS 101267	AJ292395		AF339553	DQ522406	DQ522358
*S. lanosoniveum*	CBS 704.86	AJ292396		AF339554	DQ522405	DQ522357
*S. lepidopterorum*	GY29131	MN006246			MN022273	MN022265
*S. lepidopterorum*	GY29132	MN006245			MN022274	MN022266
*S. minatense*	JCM 18176	AB603992	LC496893			
*S. minatense*	JCM 18177	AB603991				
*S. minatense*	JCM 18178	AB603993	LC496894			
*S. obclavatum*	CBS 311.74	AJ292394		AF339517		EF468798
*S. obclavatum*	JCM 18179	AB604000				
*S. spumae*	JCM 39050	LC496869	LC496898	LC496883		LC496913
*S. spumae*	JCM 39051	LC496870	LC496899	LC496884		LC496914
*S. spumae*	JCM 39054	LC496871	LC496902	LC496887		LC496917
*S. subtropicum*	JCM 18180	AB603990	LC496895			
*S. subtropicum*	JCM 18181	AB603995	LC496896			
*S. subtropicum*	JCM 18182	AB603996				
*S. subtropicum*	JCM 18183	AB604001				
*S. sympodiophorum*	JCM 18184	AB604003	LC496897			
***S. coccinellidae***	**DY101791**	**MT453861**	**MT453863**	**MT453862**		**MT471341**
***S. coccinellidae***	**DY101792**	**MT453864**		**MT457410**		**MT471342**
***S. hymenopterorum***	**DY101691**	**MT453848**	**MT453849**	**MT453850**	**MT471344**	**MT471337**
***S. hymenopterorum***	**DY101692**	**MT453851**	**MT453852**	**MT453853**		**MT471338**
***S. neolepidopterorum***	**DY101751**	**MT453854**	**MT453856**	**MT453855**		**MT471339**
***S. neolepidopterorum***	**DY101752**	**MT453857**	**MT453859**	**MT453858**		**MT471340**
***S. scarabaeoidea***	**DY101391**	**MT453842**	**MT453843**	**MT453844**	**MT471343**	**MT471335**
***S. scarabaeoidea***	**DY101392**	**MT453845**	**MT453847**	**MT453846**		**MT471336**
*Torrubiella wallacei*	CBS 101237			AY184967	EF469102	EF469073

Sequences generated in this study are shown in bold.

The datasets (*LSU* + *RPB1* + *TEF* and *SSU* + *ITS* + *LSU*) were analysis by Bayesian inference (BI) and maximum likelihood (ML) methods and aimed to analysis of the relationship among *Simplicillium* species and its related species in the family Cordycipitaceae (analysis 1) and the relationship among *Simplicillium* spp. (analysis 2), respectively. For BI, a Markov Chain Monte Carlo (MCMC) algorithm was used to generate phylogenetic trees with Bayesian probabilities using MrBayes v.3.2^34^ for the combined sequence datasets. The model for BI analysis was selected by ModelFinder^35^ in the software PhyloSuite^36^. The Bayesian analysis resulted in 20,001 trees after 10,000,000 generations. The first 4000 trees, representing the burn-in phase of the analyses, were discarded, while the remaining 16,001 trees were used for calculating posterior probabilities in the majority rule consensus tree. After the analysis was finished, each run was examined using the program Tracer v1.5^37^ to determine burn-in and confirm that both runs had converged. ML analyses were constructed with RAxMLGUI^38^. The GTRGAMMA model was used for all partitions, in accordance with recommendations in the RAxML manual against the use of invariant sites. The final alignment is available from TreeBASE under submission ID: 26290 (http://www.treebase.org).

## References

[1] Zare, R. & Gams, W. A revision of *Verticillium *section Prostrata. IV. The genera *Lecanicillium *and *Simplicillium* gen. nov. *Nova Hedwigia* **73**, 1–50 (2001).

[2] Zare R, Gams W (2008). A revision of the *Verticillium fungicola* species complex and its affinity with the genus *Lecanicillium*. Mycol. Res..

[3] Okane, I., Nonaka, K., Kurihara, Y., Abe, J. P. & Yamaoka, Y. A new species of *Leptobacillim*, *L. symbioticum*, isolated from mites and sori of soybean rust. *Mycoscience* **61**, 165–171 (2020).

[4] Wang YB (2020). Multigene phylogeny of the family Cordycipitaceae (Hypocreales): New taxa and the new systematic position of the Chinese cordycipitoid fungus *Paecilomyces hepiali*. Fungal Divers..

[5] Nonaka K, Kaifuchi S, Ōmura S, Masuma R (2013). Five new *Simplicillium* species (Cordycipitaceae) from soils in Tokyo, Japan. Mycoscience.

[6] Gomes AA (2018). *Simplicillium coffeanum*, a new endophytic species from Brazilian coffee plants, emitting antimicrobial volatiles. Phytotaxa..

[7] Crous PW (2018). Fungal Planet description sheets: 785–867. Persoonia.

[8] Zhang ZF (2017). Culturable mycobiota from Karst caves in China, with descriptions of 20 new species. Persoonia.

[9] Liu F, Cai L (2012). Morphological and molecular characterization of a novel species of *Simplicillium* from China. Cryptogam Mycol..

[10] Liang X (2016). Eight linear peptides from the deep-sea-derived fungus *Simplicillium obclavatum* EIODSF 020. Tetrahedron.

[11] Gonçalves VN (2017). Taxonomy, phylogeny and ecology of cultivable fungi present in seawater gradients across the Northern Antarctica Peninsula. Extremophiles.

[12] Shentu XP, Xiao Y, Cao ZY, Fan JX, Yu XP (2020). Comparative analysis of the diversity of the microbial communities between non-fertilized and fertilized eggs of brown planthopper, *Nilaparvata lugens* Stål. Insects.

[13] Zhang Q (2014). Diversity and biocontrol potential of endophytic fungi in *Brassica napus*. Biol. Control.

[14] Erper, İ., Saruhan, İ., Akça, İ., Aksoy, H. M. & Tuncer, C. Evaluation of some entomopathogenic fungi for controlling the green shield bug, *Palomena prasina* L. (Heteroptera: Pentatomidae). *Egypt J. Biol. Pest Control* **26**, 573–578 (2016).

[15] Shinn TS (2017). Development of a biofungicide using a mycoparasitic fungus *Simplicillium lamellicola* BCP and its control efficacy against gray mold diseases of tomato and ginseng. Plant Pathol. J..

[16] Dong QL, Dong RZ, Xing XY, Li YK (2017). A new antibiotic produced by the cyanobacterium-symbiotic fungus *Simplicillium lanosoniveum*. Nat. Prod. Res..

[17] Yu YT, He SH, Zhao QM (2013). Isolation and identification of matrine-producing fungal endophytes from *Sophora alopecuroides* in Ningxia. Sci. Agric. Sin..

[18] Wang N, Xie YP, Fan JH (2016). Pathogenicity of *Simplicillium lanosoniveum* TYL001 isolated from *Pseudaulacaspis pentagona*. Mycosystema.

[19] Gauthier NW (2014). Mycoparasitism of *Phakopsora pachyrhizi*, the soybean rust pathogen, by *Simplicillium lanosoniveum*. Biol. Control.

[20] Chen WH, Liu C, Han YF, Liang JD, Liang ZQ (2019). Three novel insect-associated species of *Simplicillium* (Cordycipitaceae, Hypocreales) from southwest china. Mycokeys.

[21] Zou X, Liu AY, Liang ZQ, Han YF, Yang M (2010). *Hirsutella liboensis*, a new entomopathogenic species affecting Cossidae (Lepidoptera) in China. Mycotaxon.

[22] Liang JD (2011). Optimal culture conditions for keratinase production by a novel thermophilic *Myceliophthora thermophila* strain GZUIFR-H49-1. J. Appl. Microbiol..

[23] White TJ, Bruns T, Lee S, Taylor J, Innis MA, Gelfand DH, Sninsky JJ, White TJ (1990). Amplification and direct sequencing of fungal ribosomal RNA genes for phylogenetics. PCR Protocols: A Guide to Methods and Applications.

[24] Rakotonirainy MS, Cariou ML, Brygoo Y, Riba G (1994). Phylogenetic relationships within the genus *Metarhizium* based on 28S rRNA sequences and isozyme comparison. Mycol. Res..

[25] Castlebury LA, Rossman AY, Sung GH, Hyten AS, Spatafora JW (2004). Multigene phylogeny reveals new lineage for *Stachybotrys chartarum*, the indoor air fungus. Mycol. Res..

[26] van den Brink J, Samson RA, Hagen F, Boekhout T, de Vries RP (2012). Phylogeny of the industrial relevant, thermophilic genera *Myceliophthora* and *Corynascus*. Fungal Divers..

[27] Mongkolsamrit S (2018). Disentangling cryptic species with Isaria-like morphs in Cordycipitaceae. Mycologia.

[28] Wei DP, Wanasinghe DN, Hyde KD, Mortimer PE, To-Anun C (2019). The genus *Simplicillium*. Mycokeys.

[29] Kondo N, Iwasaki H, Tokiwa T, Ōmura S, Nonaka K (2020). *Simplicillium spumae* (Cordycipitaceae, Hypocreales), a new hyphomycetes from aquarium foam in Japan. Mycoscience.

[30] Katoh K, Standley DM (2013). MAFFT multiple sequence alignment software version 7: Improvements in performance and usability. Mol. Biol. Evol..

[31] Tamura, K., Stecher, G., Peterson, D., Filipski, A. & Kumar, S. MEGA6: Molecular evolutionary genetics analysis version 6.0. *Mol. Biol. Evol.* **30**, 2725–2729 (2013). 10.1093/molbev/mst197 PMC3840312 24132122

[32] Vaidya G, Lohman DJ, Meier R (2011). SequenceMatrix: Concatenation software for the fast assembly of multi-gene datasets with character set and codon information. Cladistics.

[33] Swofford, D. L. *PAUP* 4.0b10: Phylogenetic Analysis Using Parsimony (*and Other Methods)*. (Sinauer, 2002).

[34] Ronquist, F. *et al*. MrBayes 3.2: Efficient Bayesian phylogenetic inference and model choice across a large model space. *Syst. Biol.* **61**, 539–542 (2012). 10.1093/sysbio/sys029 PMC3329765 22357727

[35] Kalyaanamoorthy S, Minh BQ, Wong TK, Von Haeseler A, Jermiin LS (2017). ModelFinder: Fast model selection for accurate phylogenetic estimates. Nat. Methods.

[36] Zhang D (2020). PhyloSuite: An integrated and scalable desktop platform for streamlined molecular sequence data management and evolutionary phylogenetics studies. Mol. Ecol. Resour..

[37] Drummond A, Rambaut A (2007). BEAST: Bayesian evolutionary analysis by sampling trees. BMC Evol. Biol..

[38] Silvestro D, Michalak I (2012). raxmlGUI: A graphical front-end for RAxML. Org. Divers. Evol..

[39] Jeewon R, Hyde KD (2016). Establishing species boundaries and new taxa among fungi: Recommendations to resolve taxonomic ambiguities. Mycosphere.

[40] Sung GH (2007). Phylogenetic classification of *Cordyceps* and the clavicipitaceous fungi. Stud. Mycol..

[41] Kepler RM (2017). A phylogenetically-based nomenclature for Cordycipitaceae (Hypocreales). IMA Fungus..

[42] Spatafora JW, Sung GH, Sung JM, Hywel-Jones NL, White JF (2007). Phylogenetic evidence for an animal pathogen origin of ergot and the grass endophytes. Mol. Ecol..

[43] Fukuda T, Sudoh Y, Tsuchiya Y, Okuda T, Igarashi Y (2014). Isolation and biosynthesis of preussin B, a pyrrolidine alkaloid from *Simplicillium lanosoniveum*. J. Nat. Prod..

[44] Yan, B. *et al*. A new minor diketopiperazine from the sponge-derived fungus *Simplicillium* sp. YZ-11. *Nat. Prod. Res. ***29**, 2013–2017 (2015). 10.1080/14786419.2015.1027890 25835596

[45] Huang Z, Yan SZ, Chen SL (2015). Optimization on fermentation conditions of *Simplicillium obclavatum* YX016 for the production of anthraquinones. Food Sci. Technol..

[46] Takata, K. *et al*. Aogacillins A and B produced by *Simplicillium* sp. FKI-5985: New circumventors of *Arbekacin resistance* in MRSA. *Org. Lett.* **15**, 4678–4681 (2013). 10.1021/ol401975z 24004199

[47] Uchida R (2019). Simpotentin, a new potentiator of amphotericin B activity against *Candida albicans*, produced by *Simplicillium minatense* FKI-4981. J. Antibiot..

[48] Ward NA, Robertson CL, Chanda AK, Schneider RW (2012). Effects of *Simplicillium lanosoniveum* on *Phakopsora pachyrhizi*, the soybean rust pathogen, and its use as a biological control agent. Phytopathology.

[49] Zhao D (2013). *Simplicillium chinense*: A biological control agent against plant parasitic nematodes. Biocontrol Sci. Technol..

[50] Chen RS, Huang CC, Li JC, Tsay JG (2017). Evaluation of characteristics of *Simplicillium lanosoniveum* on pathogenicity to aphids and in vitro antifungal potency against plant pathogenic fungi. Int. J. Environ. Agric. Res..

